# Kane’s Elements of Chemistry

**Published:** 1842-11

**Authors:** 


### THE WESTERN JOURNAL.

Vol. VI.—No. V.

LOUISVILLE, NOVEMBER 1, 1842.

### KANE'S ELEMENTS OF CHEMISTRY.

Professor Draper has rendered an acceptable service to American students of Chemistry, by giving them this edition of Dr. Kane's admirable work, with alterations, adapting it better to the system of instruction pursued in this country. We have no hesitation in agreeing with the editor, that, as a text-book, it is the best work extant in the English language. It represents the present condition of chemical science, and exhibits its applications to pharmacy, medicine, and the useful arts. In a work of so great detail much is given in which the student can feel but little interest. Dr. Draper has had the details printed in a smaller type, by which the learner will be able to distinguish the parts embracing the leading principles upon which he will bestow his first attention. The mechanical execution of the work is excellent. It contains a great number of wood cuts, which will materially aid the student in comprehending processes. So rapid is the march of chemical science, especially of organic chemistry, that it must be posted up at short intervals. New publications are demanded every year to bring up the improvements. Kane's Elements embrace an account of many which are not to be met with in previous works on chemistry, and it will be found that he has adapted his work admirably to the wants of medical men, by pointing out the connexion, where any existed, between his subject and pharmacy, physiology, or pathology. Y.

